# Theory-based Trial to Promote Uptake and Sustained Use of Face Coverings During the COVID-19 Pandemic

**DOI:** 10.1093/abm/kaad039

**Published:** 2023-09-01

**Authors:** Christopher J Armitage, Chris Keyworth, Nicola Gartland, Anna Coleman, David Fishwick, Sheena Johnson, Martie van Tongeren

**Affiliations:** Manchester Centre for Health Psychology, School of Health Sciences, University of Manchester, Manchester, UK; Manchester University NHS Foundation Trust, Manchester Academic Health Science Centre, Manchester, UK; NIHR Greater Manchester Patient Safety Research Collaboration, University of Manchester, Manchester, UK; Department of Psychology, University of Leeds, Leeds, UK; Centre for Occupational and Environmental Health, School of Health Sciences, University of Manchester, Manchester, UK; Manchester Academic Health Science Centre, Manchester, UK; Centre for Occupational and Environmental Health, School of Health Sciences, University of Manchester, Manchester, UK; Manchester Academic Health Science Centre, Manchester, UK; Centre for Occupational and Environmental Health, School of Health Sciences, University of Manchester, Manchester, UK; Alliance Manchester Business School, University of Manchester, Manchester, UK; Centre for Occupational and Environmental Health, School of Health Sciences, University of Manchester, Manchester, UK; Manchester Academic Health Science Centre, Manchester, UK

**Keywords:** COVID-19, Adherence, Intervention, SARS-CoV-2, Face covering, Face mask, COM-B

## Abstract

**Background:**

Transmission of airborne viruses can be mitigated by wearing face coverings but evidence suggests that face covering declines with the removal of relevant legislation, partly due to low automatic motivation.

**Purpose:**

Test whether an intervention based on implementation intentions could support people’s automatic motivation and promote face covering during the COVID-19 pandemic.

**Methods:**

Randomized controlled design. At baseline (May 20 to June 6, 2022), 7,835 UK adults reported how much time they spent wearing face coverings in work, public transport, and indoor leisure settings as well as their capabilities, opportunities, and motivations. 3,871 participants were randomized to form implementation intentions; 3,964 control participants completed questionnaires only. Measures were repeated 6 months postbaseline (November 1 to November 14, 2022). Data were analyzed using mixed measures ANOVAs and Bayes Factors to examine whether the observed data supported the experimental hypothesis.

**Results:**

The proportion of time spend wearing face coverings declined substantially across the 6-month study period, from 15.28% to 9.87% in work settings, 38.31% to 24.55% on public transport, and 9.58% to 7.85% in leisure settings. Bayes Factors indicated moderate relative evidence of no effect of implementation intentions on behavior in work and leisure settings, and inconclusive evidence of a positive effect on public transport.

**Conclusions:**

In the context of declining COVID-19 rates and removal of legal mandates, implementation intentions were not effective in sustaining face covering. Further research is required to ensure that evidence-based interventions are prepared and deployed in the event of future pandemics.

## Teaser

A brief, theory-based intervention designed to change people's habits showed a small positive effect on wearing face coverings in public transport settings

## Background

The wearing of face coverings decreases community transmission of airborne viruses including SARS-CoV-2 [1, 2]. At the height of the SARS-CoV-2 pandemic, governments in the UK made it a legal requirement to wear a face covering in defined public areas. For example, from June 15, 2020, the wearing of face coverings on public transport was a legal requirement enforceable by fines of up to £6,400 (US$8,700) [3]. Over time, these restrictions were removed and government messaging shifted from mandate to recommendations to advice. The SARS-CoV-2 pandemic is unlikely to be the last novel airborne viral threat and it would be valuable to know what would promote the uptake and sustained use of face coverings in the future. The aim of the present study was to take the learning from previous research on face covering to test a theory-based intervention to promote uptake and sustained use of face coverings.

The starting point for a theory-based intervention is identifying what needs to change [4], and there have been numerous studies that have identified predictors of adherence to requirements/guidelines for wearing face coverings. For example, using Ajzen’s [5] theory of planned behavior as a theoretical framework, Sun et al. [6] showed that attitudes and perceptions of control were predictive of students’ intentions to wear face coverings [7]. However, the theory of planned behavior has been criticized for focusing on reflective motivation (e.g., attitudes, perceptions of control) to the neglect of automatic influences on behavior, such as habits and emotions [8]. In contrast, Michie et al.’s [4] capabilities, opportunities, and motivations model of behavior (COM-B) is designed to capture all the key drivers of human behavior, including the influence of automatic motivation.

Previous studies have used COM-B, endorsed by the UK National Institute for Health and Care Excellence as a key theoretical framework for understanding and supporting behavior change [9], as a lens with which to understand the wearing of face coverings. For example, Armitage et al. [10] showed that COM-B consistently predicted people’s adherence to the wearing of face coverings in work, public transport, and indoor leisure settings. Across each of these contexts, lack of automatic motivation was a recurring finding and Armitage et al. [10] recommended focusing on interventions designed to support automatic motivation. The question then arises as to what kind of intervention could be deployed to support automatic motivation; implementation intentions [11] offer one possible solution.

Implementation intentions [11] are “if-then” plans that have been shown to impact people’s automatic motivation (e.g., habits) through which sustained changes in behavior are achieved (e.g., [12–14]). Implementation intentions are formed by asking people to link critical situations (“if”) with appropriate responses (“then”). The effect of implementation intention formation on automatic motivation is demonstrated through laboratory studies which show that specifying the “if” component of an implementation intention enhances the accessibility of critical situations and that linking “if” with “then” automates the response specified in the “then” component [13]. For example, one possible cue might be “being tempted not to wear a face covering consistently” and it could be linked to “seeking out someone who encourages me to wear a face covering when I don’t feel up to it” as an appropriate response. The idea is that if the temptation not to wear a face covering is encountered, the appropriate response (“seeking out someone who encourages me to wear a face covering when I don’t feel up to it”) is triggered automatically. Thus, implementation intentions are one means by which automatic motivation can be changed to promote sustained behavior change [12, 13].

Several meta-analyses now attest to the effectiveness of implementation intention-based interventions in areas such as smoking cessation [14], but not in relation to face covering. However, Gollwitzer and Sheeran’s [13] meta-analysis across multiple behavioral domains showed that, across 94 independent studies in laboratory and field settings, implementation intention-based interventions exerted an average effect size of *d* = 0.65. We were unable to identify previous research applying implementation intentions to the problem of face covering, but conclude there are promising grounds for pursuing this approach.

### The Current Study

For the first time, the present study aims to test an implementation intention-based intervention to promote uptake and sustained use of face coverings in large samples that are representative of the UK population, and to understand changes using COM-B. Based on the research reviewed above, it is predicted that participants randomized to form implementation intentions will have higher automatic motivation to wear a face covering and will be more likely to wear a face covering.

## Method

### Study Design

This was a randomized controlled trial and had a mixed design. The between-persons factor was *condition*, which had two levels: *Intervention* in which participants were asked to form implementation intentions and *control* in which participants were not asked to form implementation intentions. All dependent measures were taken at baseline and follow-up meaning that the within-persons factor was *time*. Follow-up occurred 6 months postbaseline, a time period that is commonly regarded as the period by which behavior change is considered maintained [15]. The principal outcome measure was the proportion of time wearing face coverings, the other dependent variables were people’s capabilities, opportunities, and motivations to wear a face covering. The trial was preregistered.

### Recruitment and Participants

YouGov, a market research company, recruited a sample of 7,835 UK residents aged 18+ that was designed to be representative of the UK adult population. YouGov have a database of more than 1 million potential participants and participants were incentivized in line with YouGov’s points system. The data were sent securely to the research team for analysis. Ethical approval was obtained from a Research Ethics Committee and participants gave informed consent at the beginning of the survey. The baseline characteristics of the sample are presented in Table 1.

**Table 1:** Baseline Sociodemographic Characteristics of the Sample

Variable	Intervention (*N* = 3,871)	Control (*N* = 3,964)
Gender		
Men	48.9%	48.1%
Women	51.1%	51.9%
Age	*M* = 50.0 *SD* = 17.2	*M* = 50.0 *SD* = 17.1
Social grade		
Nonmanual	51.1%	50.5%
Manual/unemployed	48.9%	49.5%
Ethnicity		
Asian	5.6%	4.6%
Black	1.8%	1.5%
Mixed identity	2.5%	2.9%
White	89.1%	90.4%
Any other ethnic identity/Prefer not to say	1.0%	0.6%
Country		
England	84.3%	83.8%
Northern Ireland	2.8%	2.9%
Scotland	8.0%	8.5%
Wales	4.9%	4.8%

### Instrument

#### Sociodemographic variables

Measures of age, gender, ethnicity, social grade, and country (i.e., England, Northern Ireland, Scotland, Wales) were taken using standard UK Office for National Statistics [16] items.

#### Behavior

Participants rated the extent to which they wore face coverings on 0%–100% scales using the items, “Of the time you spent *at work/on public transport/ doing leisure activities that brought you into contact with other people in indoor spaces (e.g., cinemas, theatres, live music, nightclubs)* in the last 7 days, roughly what percentage of it did you spend wearing a face covering?”

#### Psychosocial variables

Keyworth et al.’s [17] COM-B measure was used to assess people’s capabilities, opportunities, and motivations with respect to wearing face coverings at work, on public transport, and during leisure activities. Capabilities are further subdivided into physical capability (e.g., having appropriate skills) and psychological capability (e.g., having the requisite knowledge); opportunities into physical opportunity (e.g., sufficient time) and social opportunity (e.g., supportive colleagues); and motivations into automatic motivation (e.g., habits) and reflective motivation (e.g., consciously planning to do something).

The items are based on Keyworth et al.’s [17] measure that comprises six items designed to tap physical capability, psychological capability, physical opportunity, social opportunity, reflective motivation, and automatic motivation, which are presented in Tables 2–4. The items are accompanied by brief definitions of each of the constructs (e.g., the reflective motivation item is accompanied with: “What is motivation? Conscious planning and evaluation (beliefs about what is good and bad) (e.g., I have the desire to, I feel the need to)).

**Table 2:** Effects of the Intervention on Face Covering at Work

	Baseline		Follow-up		*M*_difference in change over time_ (*SE*; 95%CI)	*F*	*n*_p_^2^	Bayes Factors
Variables	*M*	*SD*	*M*	*SD*				
Face Covering (0%–100%)					−0.05 (0.73; −1.48, 1.38)	0.07	<0.01	0.07
Intervention, *n* = 1,797	15.43	30.97	9.92	25.58				
Control, *n* = 1,839	15.14	30.77	9.82	25.53				
Physical capability: “I am PHYSICALLY able to wear a face covering at work” (0–10)					0.08 (0.08; −0.08, 0.24)	1.02	<0.01	0.22
Intervention, *n* = 1,797	7.65	2.91	7.60	2.97				
Control, *n* = 1,839	7.80	2.85	7.67	2.95				
Psychological capability: “I am PSYCHOLOGICALLY able to wear a face covering at work” (0–10)					0.08 (0.09; −0.09, 0.25)	1.27	<0.01	0.21
Intervention, *n* = 1,797	7.02	3.14	6.97	3.14				
Control, *n* = 1,839	7.17	3.06	7.01	3.13				
Physical opportunity: “Of the time you spent working in the last 7 days, roughly what percentage of it did you have the PHYSICAL opportunity to wear a face covering?” (0%−100%)					−1.47 (1.26; −3.93, 0.99)	1.31	<0.01	0.06
Intervention, *n* = 1,797	69.17	42.11	66.14	43.88				
Control, *n* = 1,839	66.81	42.94	65.20	43.85				
Social opportunity: “Of the time you spent working in the last 7 days, roughly what percentage of it did you have the SOCIAL opportunity to wear a face covering?” (0%–100%)					−0.10 (1.70; −3.45, 3.24)	0.01	<0.01	0.16
Intervention, *n* = 1,797	54.33	45.43	61.99	44.78				
Control, *n* = 1,839	52.84	45.36	60.60	44.84				
Reflective motivation: “I am motivated to wear a face covering at work” (0–10)					0.06 (0.08; −0.10, 0.21)	0.42	<0.01	0.16
Intervention, *n* = 1,797	3.82	3.40	3.30	3.19				
Control, *n* = 1,839	3.89	3.42	3.33	3.23				
Automatic motivation: “Wearing a face covering at work is something that I do automatically” (0–10)					0.04 (0.08; −0.12, 0.21)	0.26	<0.01	0.12
Intervention, *n* = 1,797	3.44	3.49	2.74	3.26				
Control, *n* = 1,839	3.43	3.51	2.70	3.29				

*F* and *n*_p_^2^ refer to the test of the condition *×* time interaction.

**Table 3:** Effects of the Intervention on Face Covering on Public Transport

	Baseline		Follow-up		*M*_difference in change over time_ (*SE*; 95%CI)	*F*	*n*_p_^2^	Bayes Factors
Variables	*M*	*SD*	*M*	*SD*				
Face Covering (0%–100%)					1.22 (1.29; −1.34, 3.75)	1.00	<0.01	0.33
Intervention, *n* = 1,525	38.67	45.44	25.57	40.81				
Control, *n* = 1,565	37.95	45.75	23.55	39.69				
Physical capability: “I am PHYSICALLY able to wear a face covering on public transport” (0–10)					−0.09 (0.08; −0.24, 0.06)	1.15	<0.01	0.04
Intervention, *n* = 1,525	8.17	2.68	8.02	2.79				
Control, *n* = 1,565	8.25	2.59	8.18	2.63				
Psychological capability: “I am PSYCHOLOGICALLY able to wear a face covering on public transport” (0–10)					−0.09 (0.08; −0.25, 0.07)	1.22	<0.01	0.04
Intervention, *n* = 1,525	7.80	2.79	7.59	2.95				
Control, *n* = 1,565	7.82	2.78	7.71	2.86				
Physical opportunity: “Of the time you spent on public transport in the last 7 days, roughly what percentage of it did you have the PHYSICAL opportunity to wear a face covering?” (0%–100%)					1.40 (1.81; −2.15, 4.95)	0.65	<0.01	0.37
Intervention, *n* = 1,525	77.37	39.30	71.92	42.65				
Control, *n* = 1,565	78.39	38.80	71.48	42.75				
Social opportunity: “Of the time you spent on public transport in the last 7 days, roughly what percentage of it did you have the SOCIAL opportunity to wear a face covering?” (0%–100%)					1.23 (1.40; −1.52, 3.97)	0.87	<0.01	0.33
Intervention, *n* = 1,525	67.34	42.13	62.82	44.54				
Control, *n* = 1,565	66.69	42.78	60.85	44.73				
Reflective motivation: “I am motivated to wear a face covering on public transport” (0–10)					0.10 (0.09; −0.07, 0.27)	1.53	<0.01	0.28
Intervention, *n* = 1,525	5.43	3.68	4.65	3.56				
Control, *n* = 1,565	5.35	3.67	4.49	3.54				
Automatic motivation: “Wearing a face covering on public transport is something that I do automatically” (0–10)					0.12 (0.09; −0.07, 0.30)	1.53	<0.01	0.39
Intervention, *n* = 1,525	5.06	3.79	4.05	3.66				
Control, *n* = 1,565	4.95	3.77	3.83	3.62				

*F* and *n*_p_^2^ refer to the test of the condition *×* time interaction.

**Table 4:** Effects of the Intervention on Face Covering During Leisure Activities

	Baseline		Follow-up		*M*_difference in change over time_ (*SE*; 95%CI)	*F*	*n*_p_^2^	Bayes Factors
Variables	*M*	*SD*	*M*	*SD*				
Face covering (0%–100%)					−0.70 (0.63; −1.93, 0.53)	1.22	<0.01	0.03
Intervention, *n* = 1,528	10.38	26.18	8.30	24.00				
Control, *n* = 1,608	8.82	24.81	7.42	23.02				
Physical capability: “I am PHYSICALLY able to wear a doing leisure activities” (0–10)					0.03 (0.08; −0.14, 0.19)	0.12	<0.01	0.11
Intervention, *n* = 1,528	7.39	3.15	7.36	3.19				
Control, *n* = 1,608	7.44	3.14	7.38	3.18				
Psychological capability: “I am PSYCHOLOGICALLY able to wear a face covering doing leisure activities” (0–10)					−0.11 (0.09; −0.28, 0.06)	1.32	<0.01	0.04
Intervention, *n* = 1,528	6.91	3.25	6.80	3.35				
Control, *n* = 1,608	6.81	3.32	6.81	3.38				
Physical opportunity: “Of the time you spent doing leisure activities in the last 7 days, roughly what percentage of it did you have the PHYSICAL opportunity to wear a face covering?” (0%–100%)					1.36 (1.27; −1.12, 3.84)	1.23	<0.01	0.38
Intervention, *n* = 1,528	61.16	44.69	61.54	44.67				
Control, *n* = 1,608	61.08	44.91	60.06	45.10				
Social opportunity: “Of the time you spent doing leisure activities in the last 7 days, roughly what percentage of it did you have the SOCIAL opportunity to wear a face covering?” (0%–100%)					2.38 (1.29; −0.16, 4.92)	3.20	<0.01	1.32
Intervention, *n* = 1,528	49.25	45.10	49.39	45.55				
Control, *n* = 1,608	49.66	45.34	47.49	45.26				
Reflective motivation: “I am motivated to wear a face covering doing leisure activities” (0–10)					−0.08 (0.07; −0.22, 0.05)	1.23	<0.01	0.03
Intervention, *n* = 1,528	3.71	3.37	3.21	3.23				
Control, *n* = 1,608	3.54	3.33	3.12	3.20				
Automatic motivation: “Wearing a face covering doing leisure activities is something that I do automatically” (0–10)					−0.14 (0.07; −0.28, −0.01)	3.94	<0.01	0.02
Intervention, *n* = 1,528	3.30	3.24	2.73	3.05				
Control, *n* = 1,608	3.15	3.24	2.72	3.10				

*F* and *n*_p_^2^ refer to the test of the condition *×* time interaction.

### Intervention

In addition to completing the measures described above, participants were randomized to one of two conditions. Participants in the control condition exited the online survey after completing the baseline questionnaire. Participants in the intervention condition were additionally asked to plan to wear a face covering and were presented with a “volitional help sheet,” a tool for helping people to form implementation intentions [12]. The volitional help sheet comprised the critical situation “if I am tempted not to wear a face covering consistently,” with a drop-down menu that allowed participants to link this critical situation with up to 10 appropriate responses (see Appendix). The appropriate responses were adapted from previous volitional help sheets [12], which in turn were adapted from Prochaska and DiClemente’s [15] processes of change from their transtheoretical model. The appropriate responses included: “then I will seek out someone who encourages me to wear a face covering when I don’t feel up to it,” “then I will think about how I would be a better role model for others if I were to wear a face covering,” and “then I will put things around my home to remind me to wear a face covering.”

### Data Collection

The data were collected via online surveys in two waves. At the time of data collection, there were no legal requirements to wear face coverings in any setting. Baseline data collection was conducted May 20 to June 6, 2022 when Median new COVID-19 cases was 6,336 (4,656–33,053) per day [18]. Follow-up was conducted 6 months later, November 1 to November 14, 2022 when Median new COVID-19 cases was 0 (0–31,552) per day.

### Statistical Analyses

Data were weighted, by age, gender, social class, country of residence, and level of education to ensure analyses properly reflected the UK population. Descriptive statistics were used to characterize the population (Table 1). The analyses were conducted on an intention-to-treat basis, such that people who dropped out of the study were treated as no changers.

Randomization was tested using MANOVA. This was designed to establish that the intervention and control groups were similar in terms of their demographics and other descriptive characteristics. The principal outcome measures were tested using mixed ANOVAs. *Condition* (intervention versus control) was the between-participants factor, and *time* (baseline vs. 6-month follow-up) the within-persons factor. Proportion of time spent wearing a face covering was the main outcome measure; capabilities, opportunities, and motivations were the secondary outcome measures. An online calculator https://harry-tattan-birch.shinyapps.io/bayes-factor-calculator/ was used to examine whether the observed data supported the experimental hypothesis (a 10% improvement in COM-B scores) or the null hypothesis (no change). Bayes Factors greater than 3 indicate moderate relative evidence for an effect; Bayes Factors lower than 0.33 indicate moderate relative evidence for no effect.

## Results

### Participant Characteristics

**Fig. 1.**
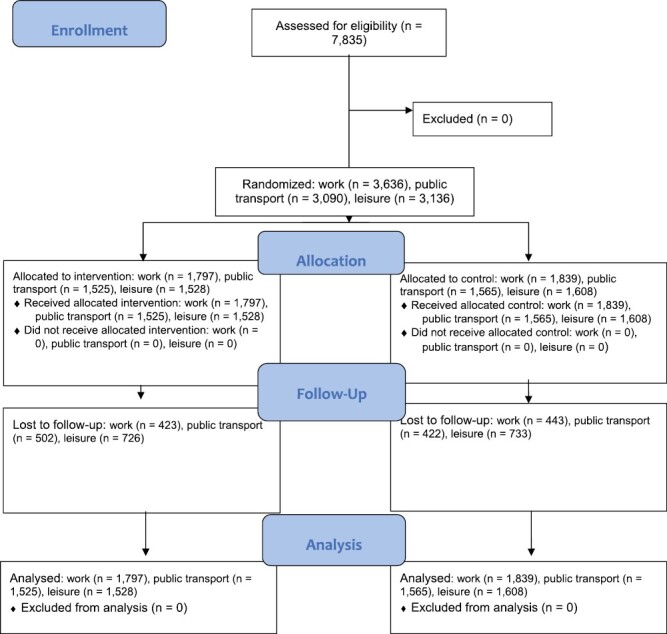
Flow of participants through the trial.

Consistent with the sampling frame, the baseline sample (*N* = 7,835, Fig. 1) was broadly representative of the UK population [16]. Comparison of sociodemographic characteristics at baseline, using MANOVA, revealed no statistically significant differences between those who were randomized to the intervention group and those randomized to the control group, *F*(5, 7,856) = 0.80, *p* = .55, *n*_p_^2^ < 0.01 (Table 1).

The wearing of face coverings declined substantially across the 6-month study period, from 15.28% to 9.87% in work settings (Table 2), 38.31% to 24.55% on public transport (Table 3), and 9.58% to 7.85% in leisure settings (Table 4). People’s perceptions of their capabilities and opportunities with respect to wearing face coverings remained stable over time, but their reflective and automatic motivation diminished substantially: Across each of the three contexts, the decreases in motivation accounted for >5% of the variance.

### Effects of the Intervention

A series of mixed ANOVAs with *condition* (intervention vs. control) as the between-participants factor, and *time* (baseline vs. 6-month follow-up) as the within-persons factor was used to test the effects of the intervention (Tables 2–4). Tests of statistical significance implied no evidence to suggest that the implementation intention-based intervention arrested the decline in the wearing of face coverings or influenced people’s perceptions of their capabilities, opportunities, and motivations in any of the three contexts.

However, examination of Bayes Factors (Tables 2–4) reveals a more nuanced picture. For face covering in work settings (Table 2), each analysis was associated with Bayes Factors lower than 0.33 indicating moderate relative evidence for no effect. In contrast, the Bayes Factors associated with several dependent variables for face covering on public transport exceeded 0.33, but did not breach 3.00, meaning inconclusive evidence of a positive effect of the intervention on public transport (Table 3). More specifically, there were positive changes over time in behavior, physical opportunity, social opportunity, and automatic motivation in relation to face covering on public transport. For example, the decline in wearing face coverings on public transport was less steep in the intervention group. For leisure settings, there was similarly moderate relative evidence for no effect of the intervention (Table 4), with the exception of physical opportunity and social opportunity, both of which showed inconclusive evidence of a positive effect of the intervention.

## Discussion

### Principal Findings

The aim of the present study was to test the potential effects of an implementation intention-based intervention on uptake and sustained use of face coverings. In the context of declining infection rates and absence of relevant legislation, the wearing of face coverings decreased over time. Over time, people’s perceptions of their capabilities and opportunities remained relatively stable compared to their motivations. An intervention based on Gollwitzer’s [11] concept of implementation intentions largely failed to address this decline, albeit with some areas of uncertainty, particularly with respect to the wearing of face coverings on public transport.

### Previous Studies

Meta-analyses show that, in general, implementation intentions are effective in changing people’s behavior over sustained periods of time [13, 14], which contrasts with the present inconclusive findings. However, in the context of a public health emergency that was abating, it is unclear whether the present study is directly comparable with previous studies of implementation intentions in relation to (for example) smoking [12] and dietary intake [19]. Indeed, smoking and dietary intake are typically presented as enduring threats whereas face covering was presented as a response to the acute threat of COVID-19, rather than the relatively enduring threat of airborne transmissible viruses.

### Implications

From the perspective of developing interventions to encourage face covering, it would be valuable to examine what would be the effect of reframing the wearing of face coverings in response to the enduring threat of airborne transmissible viruses rather than the relatively acute threat of COVID-19. It is perhaps instructive that the most promising findings were on public transport, where people are often forced to be in close proximity and lack the ventilation of many work places and leisure settings. In terms of augmenting the present intervention, there is a growing body of research showing that repeated administration of implementation intentions are particularly effective at changing people’s behavior [20, 21]; it is plausible that a single administration of implementation intentions may not have been sufficient to change this particular behavior.

### Strengths and Limitations

Although the present research takes the literature on implementation intentions forward in some important respects it is important to reflect on strengths and limitations of the study. Null findings are difficult to interpret, but the large representative samples allay concerns about statistical power. However, the self-reported outcome measure is a limitation and it would be valuable to develop objective measures of face covering.

### Future Research

The consistent finding was that motivation decreased over time, irrespective of context and so further work using models such as West and Michie’s [22] PRIME theory, which seeks to explain the interplay of reflective and automatic motivation, is required to develop interventions to promote the wearing of face coverings, should they be required in the future. Implementation intentions might be part of this solution, but may be insufficient on their own to change the wearing of face coverings.

## Conclusions

A single administration of an implementation intention-based intervention was insufficient to change people’s behavior with respect to wearing face coverings, but it would be worthwhile exploring repeated administration of implementation intentions. As infection rates declined, so too did people’s wearing of face coverings; while people’s perceptions of their capabilities and opportunities remained stable, levels of motivation declined. Preparations for future viral pandemics that are airborne transmissible should focus on motivating people to take up face coverings.

## Appendix

If I am tempted not to wear a face covering consistently then I will…

1. think about information from articles and advertisements on how to make wearing a face covering a regular part of my life
2. remember how warnings about the health hazards of not wearing a face covering move me emotionally
3. think how I would be a better role model for others if I were to wear a face covering
4. tell myself that wearing a face covering would make me a healthier, happier person to be around
5. make myself wear a face covering anyway because I know I will feel better afterward
6. tell myself that I am being good to myself by taking care of my body in this way
7. seek out someone who encourages me to wear a face covering when I don’t feel up to it
8. tell myself that society is changing in ways that make it easier for people who want to wear face coverings
9. tell myself that if I try hard enough I can keep wearing a face covering
10. put things around my home to remind me to wear a face covering
